# Effect of rubbing on the in vitro skin permeation of diclofenac-diethylamine 1.16% gel

**DOI:** 10.1186/1756-0500-5-321

**Published:** 2012-06-21

**Authors:** Nathalie Hasler-Nguyen, Grigorios Fotopoulos

**Affiliations:** 1 Novartis Consumer Health, Nyon, Switzerland

## Abstract

**Background:**

Rubbing a topical NSAID (non steroidal anti-inflammatory drug) on the skin may increase local drug permeation, affecting its distribution to the site of pain and inflammation. The present study evaluates this hypothesis, by assessing in vitro the effect on skin permeation of applying diclofenac-dieythylamine 1.16% gel with or without rubbing.

**Methods:**

A single dose of 5 mg/cm^2^ diclofenac-diethylamine 1.16% gel was applied on excised human skin mounted in Franz-type diffusion cells without or with rubbing for 45 s. Drug penetration into the skin layers was determined after 1 h using the tape stripping technique. In vitro cutaneous permeation into the receptor fluid of the diffusion chamber was measured up to 24 h. Skin electrical resistance was also recorded.

**Results:**

Application of diclofenac-diethylamine 1.16% gel with rubbing resulted to a 5-fold higher flux of diclofenac through the skin than when applied without rubbing at 8 h (*P* = 0.04). Skin rubbing for 45 s decreased by 2-fold skin electrical resistance when compared to the standard application. Application of diclofenac-diethylamine 1.16% gel with rubbing tended to result in higher accumulation in the stripped skin vs. the superficial skin layers when applied without rubbing (*P* = 0.2).

**Conclusion:**

These results suggest that rubbing may alter the superficial skin layer resulting in a transient faster initial diffusion of topically applied diclofenac through the stratum corneum into the deeper skin layer of the dermis to the tissue target.

## Background

Diclofenac is a non-steroidal anti-inflammatory drug (NSAID) with known analgesic and anti-inflammatory properties. Topical formulations of diclofenac have been developed aiming to provide an effective analgesic and anti-inflammatory effect, while reducing the risk for systemic adverse effects that can be experienced with oral NSAID formulations [1]. In its various formats (gel, spray, patch etc.) diclofenac is widely used and is clinically proven to relieve pain, treat inflammation and reduce swelling in a number of acute and chronic conditions affecting the joints and muscles [2,3] .

Topical diclofenac application produces a higher local concentration of the drug, targeting the site of inflammation and pain, where it preferentially distributes and persists, achieving sufficiently high concentration to exert local therapeutic activity, while systemic drug levels are minimal [4]. Skin absorption is one of the most critical factors for the effective distribution of the drug to the site of inflammation and thus it can affect the efficacy of the topical formulation. The route of drug penetration across the stratum corneum, which is the main rate limiting barrier to skin absorption [5], depends on the physiochemical properties of the respective drug.

There is a common perception that the act of rubbing/massaging a topical NSAID on the site of pain may provide an additional benefit to the patient. The high placebo effect observed in the clinical trials with topical NSAIDs argues in favour of this perception [1]. The mechanisms by which the act of rubbing may affect the efficacy of the drug are not clear and they have not being investigated extensively, but they may involve the activation of gating mechanisms and the desensitization of local nerve endings. With this present study, we sought to assess the hypothesis that rubbing a topical NSAID onto the skin may result in increased penetration of the drug through the skin layer and thus affecting the availability of the active ingredient in subcutaneous tissues. As the permeability properties of the stratum corneum remain unchanged after removal from the body and a good correlation has been observed between in vivo and in vitro experiments with the same drug [6-8], an in vitro diffusion through non viable human skin has been considered a convenient experimental model to evaluate the permeation characteristics of a topical drug formulation. Thus, the present study compared the in vitro skin permeation and distribution of 1.16% diclofenac-diethylamine gel after application without or with a rubbing for 45 s.

## Methods

### Product

Voltaren Emulgel (Novartis Consumer Health, Nyon Switzerland) containing 1.16% diclofenac-diethylamine.

### Product application

A single dose of 5 mg/cm^2^ was applied on excised human skin mounted in Franz-type diffusion cells under non-occlusive conditions. Rubbing was performed for 45 s using the rounded end of a syringe piston while standard application (considered as “without rubbing” under these experimental conditions) of product onto skin lasted 5 s.

### Skin donor

The ethical committee of the International Institute for the Advancement of Medicine (IIAM; United States) approved the use of human tissue in this study. Full thickness human abdominal skin from 6 donors, which was taken at autopsy, was provided cryopreserved by IIAM. The skin was maintained frozen at −80°C. Prior to use, the skin was thawed and the subcutaneous tissue was carefully removed. The skin was dermatomed to 600 μm using a Wagner dermatome (model GB-231 Aesculap, Germany) to obtain split thickness tissue constituted of the stratum corneum (10–20 μm), the epidermis (100 μm) and part of the dermis (1200 μm) [9,10].

### Test of skin integrity

The permeation of tritiated water was evaluated to determine the skin integrity as described by Bronaugh et al. [11]. Briefly, tritiated water (2.7 μCi/ml) was applied to the skin surface. After 30 min, the radiolabelled water was removed from the skin with cotton-wool tips. The amount of tritiated water (%) that permeated across the skin was measured in the receptor phase (2 ml), using a liquid scintillation counter. Less than 1% of the applied dose of tritiated water permeated through the skin. Skin with tritiated water permeation higher than 1% was excluded from the experiment. The experimental procedure was performed with skin samples having similar tritiated water permeation values.

### Skin permeation

Skin permeation was measured using a static Franz diffusion cell of 1.75 cm^2^ for each skin sample exposed to 5 mg/cm^2^ of the product in accordance to the test guideline OECD 428 [12].

Thawed human dermatomed (600 μm) skin samples were mounted horizontally on the Franz cells with the dermis facing down. The Franz diffusion cells were connected to a circulating water bath at 37°C, which yielded a tissue temperature of 32°C, comparable to the physiological temperature of the skin surface. The diffusion area was 1.75 cm^2^ and the receptor phase of PBS pH 7.4 (phosphate buffered saline; 7.58 g/L Na_2_HPO_4_, 1.62 g/L NaH_2_PO_4_ and 4.4 g/L NaCl) contained within each diffusion cell (approximately 8 ml) was mixed using a magnetic stirring device to ensure appropriate homogenization of the released drug in the acceptor phase throughout the experiment. Samples of the receptor phase were collected and analysed to determine diclofenac concentration at baseline, 2 h, 4 h, 8 h and 24 h (Table 1).

**Table 1:** Comparison of drug flux from gel

**Flux (μg/cm**^**2**^**/h)**	**Diclofenac 1.16% gel applied without rubbing (R)**		**Diclofenac 1.16% gel applied with rubbing (T)**		**Ratio (T/R)**
Time (h)	Mean	s.e.m	Mean	s.e.m	
0	0.000	0.000	0.000	0.000	0.0
2	0.000	0.000	0.000	0.000	0.0
4	0.000	0.000	0.000	0.000	0.0
8	0.003*	0.003	0.018*	0.007	5.2*
24	0.027	0.006	0.035	0.007	1.3

Values represent mean from 11 replicates with standard error of mean (s.e.m), (* *P* < 0.05).

### Cutaneous distribution

Cutaneous distribution was determined by measuring the amount of drug residing in the various skin layers. One hour (1 h) after the application of 5 mg/cm^2^, skin samples were washed off with soapy water and cotton-wool tips. The top layer of the stratum corneum was removed with adhesive tape (3 M Scotch n° 550) and analyzed separately. Additional layers of the stratum corneum were removed up to 12 consecutive adhesive tape strips.

Following collection of all strips, the first and the second strips were put into separate vials containing 10 ml of water. Strips 3 up to 7 were pooled in the same vial containing 20 ml of water. The remaining stripped skin was minced and placed into a vial containing 15 ml PBS pH 7.4. Mixtures were stirred overnight to ensure adequate extraction of the drug from the tape and the skin.

### Sample analysis

Diclofenac content in the various samples was measured by ultra high performance liquid chromatography (Waters Acquity) on a Waters Aquity UPLC®BEH C18 1.7 μm (2.1 × 50 mm) phase column at 35°C using a flow rate of 0.5 ml/min of the mobile phase of a mixture of purified water with 1% acetic acid and acetonitrile 4.2:5.8 (v:v) with UV detection at 275 nm. The sample was directly injected at a volume of 10 μl. The retention time of diclofenac was 3.2 min. The area under the peak was used to calculate the concentration of diclofenac using external standards that showed a linearity over the concentration range of 0.03 to 1 μg/ml.

### Measurement of tissue resistance

Discs of excised human abdominal split thickness skin of 600 μm were put on Transwell insert without membrane. The diffusion area was 1.1 cm^2^ and the receiver phase consisted of PBS pH 7.4 at 32°C (1.5 ml) with a donor phase of 0.5 ml PBS pH 7.4 at 32°C. Skin resistance was measured before rubbing. After the pretreatment 0.5 ml of donor phase preheated at 32°C was added. After 5 min up to 2 h the tissue resistance was determined with EVOMX (World Precision Instruments, Sarasota, FL). Ratio of the resistance before and after the rubbing were calculated and compared to the same non-rubbed skin.

### Statistical analysis

Unpaired Student *t*-test were performed to define a *P* value between applications with or without rubbing. A *P* value of less than 0.05 was considered statistically significant.

## Results

**Figure 1:**
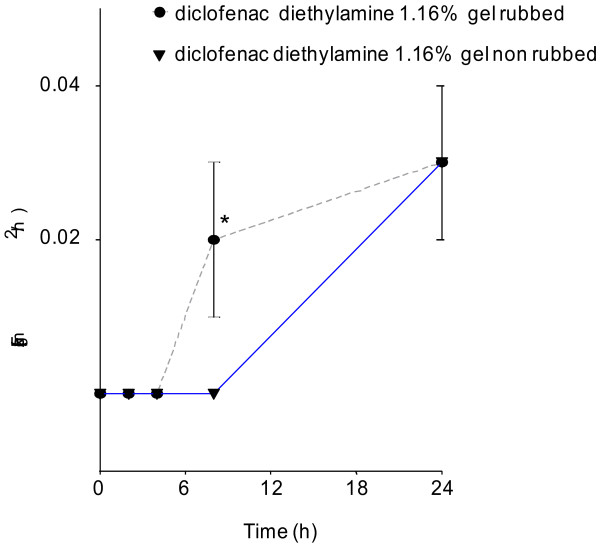
**Comparison of diclofenac flux after application of diclofenac-diethylamine 1.16% gel applied with or without rubbing.** Values represent mean from 11 replicates with mean standard errors (* *P* < 0.05).

Diclofenac flux after application of diclofenac-diethylamine 1.16% gel without and with rubbing for 45 s is shown in Figure 1 and Table 1. When the product was applied on the skin followed by rubbing the flux was 5-fold higher at 8 h than when applied without rubbing (*P* = 0.04). This difference leveled off at the end of the experiment at 24 h.

**Figure 2:**
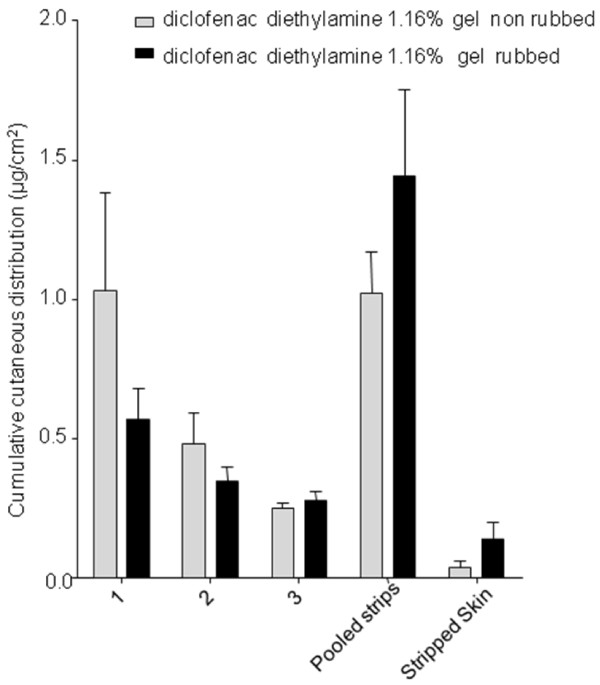
**Comparison of skin electrical resistance with or without rubbing.** Values represent mean from at least 5 replicates with mean standard errors (*** *P* < 0.001).

To further evaluate the effect of rubbing on the skin barrier, the trancutaneous electrical resistance was measured (Figure 2). The transcutaneous electrical resistance values at 5 min and 2 h were similar and, thus, only the 2 h data are shown in Figure 2. The electrical resistance before rubbing was between 6 kΩ/cm^2^ – 20 kΩ/cm^2^ and after rubbing between 4 kΩ/cm^2^ – 13 kΩ/cm^2^. Thus the skin transcutaneous electrical resistance tended to be 2-fold lower after application of the product with rubbing than without (*P* < 0.001).

**Figure 3:**
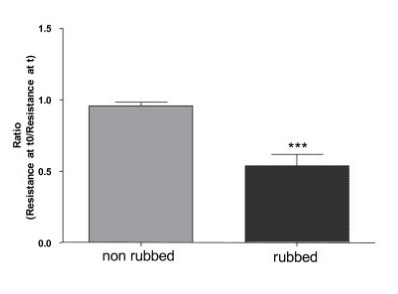
**Comparison of diclofenac’s concentration in individual tape strips (1,2,3), in the pooled strips and in the remaining stripped skin after application of diclofenac-diethylamine 1.16% gel with or without rubbing.** Values represent mean from 11 replicates with mean standard errors.

Finally, application of diclofenac-diethylamine 1.16% gel with a 45 s rubbing tended to result in higher accumulation in the stripped skin (*P* = 0.2; Figure 3). In contrast, it appears that when diclofenac-diethylamine 1.16% gel was applied without rubbing it tended to remain in the superficial skin layers, as represented by strip 1 in Figure 3 (*P* = 0.2).

## Discussion

This in vitro study demonstrated that rubbing diclofenac-diethylamine 1.16% gel for 45 s resulted in a 5-fold higher flux of diclofenac through the skin when compared to application without rubbing. This effect was observed at 8 h post application, while concentration of the active penetrating through the skin reached a plateau after 24 h. This indicates that the act of rubbing may affect the speed of the transcutaneous penetration of the drug through the skin, which may result in transient higher availability of the active in the subcutaneous muscle and joint tissues.

A consistent trend was also observed between the kinetics of diclofenac absorption through the skin and its electrical resistance when the product was applied with rubbing. Such application, in addition to increasing diclofenac’s flux, also resulted to a 2-fold reduction of the skin’s electrical resistance. Furthermore, application with rubbing resulted in numerically higher concentration of diclofenac in stripped skin, corresponding to the deeper skin layer.

These findings suggest that the act of rubbing may transiently affect skin’s lipid structure, resulting in a facilitated penetration through the skin layers. However, the rubbing did not seem to disrupt the skin barrier. Indeed, the electrical resistance of the skin explants used in this in vitro study, was constantly higher than 3.9 kΩ/cm^2^ which is the cut-off value for considering the skin intact [13]. Further experiments may be needed to evaluate the effect of rubbing on the skin hydration, as permeability is known to depend on the water content of the stratum corneum [14]. It is believed that hydration induces stratum corneum swelling and fluidization of lipid bilayers leading to a reduction of the electrical resistance [14], which was observed in this study.

Measurement of in vitro permeation across the split thickness of human skin allows the evaluation of the passive diffusion of the molecule into and across the skin to a solute reservoir, which simulates the capillaries of the dermis. As permeability properties of the stratum corneum remain unchanged in non-viable human skin the results of this study may be extrapolated to in-use condition. Several factors can influence a drug skin permeation, such as its release from the formulation, its penetration into the stratum corneum layers (10–20 μm) and its diffusion through the stratum corneum into the various epidermis layers (100 μm) and the dermis (1200 μm). The in vitro system used in this study provided an insight on the kinetics of diclofenac through the skin and on the role that the act rubbing may play in altering the skin layer parameters, to allow the drug to reach the subcutaneous tissues faster, increasing their initial exposure to it. This may potentially affect the efficacy of the drug and may partially explain the positive effect of rubbing/massaging observed in the clinical setting with the use of topical NSAIDs. Further studies, and particularly prospective human clinical trials, will be needed to further evaluate the findings demonstrated with this in vitro model.

## Conclusions

Application of diclofenac-diethylamine 1.16% gel with rubbing for 45 s increased diclofenac flux through the skin up to 5-fold at 8 h. However, at 24 h, the amount of diclofenac penetrating through the skin after standard application or application with rubbing was not different. This indicates that the act of rubbing may accelerate the speed of the drug transcutaneous delivery without altering the integrity of the skin.

This in vitro study demonstrated for the first time that the flux of a molecule across the skin can be modified by a mechanical act. In general gel formulations are applied by rubbing, but this study demonstrated that the duration of this action may play an important role and prolonging it may result to a higher flux of the drug through this skin barrier. Since the properties of the stratum corneum remain unchanged when it is removed from the body [6-8] these in vitro observations may correlate well with the in vivo situation. Further clinical studies are required to confirm this hypothesis.

## Competing interests

This study was funded by Novartis Consumer Health. NHN and GF are employees of Novartis Consumer Health. Both authors declare they have no other competing interests.

## Authors’ contributions

NHN contributed to the analysis, interpreted results and drafted manuscript. GF have contributed to the development of the project’s concept and critically edited the manuscript. All authors read and approved the final manuscript.
